# Relationships between erythrocyte membrane mono- and poly- unsaturated fatty acid composition and clinical/cognitive indices in antipsychotic-free schizophrenia patients

**DOI:** 10.3389/fpsyt.2024.1361997

**Published:** 2024-04-23

**Authors:** Yuko Higuchi, Tsutomu Takahashi, Hiroko Itoh, Daiki Sasabayashi, Tomiki Sumiyoshi, Michio Suzuki

**Affiliations:** ^1^ Department of Neuropsychiatry, University of Toyama Graduate School of Medicine and Pharmaceutical Sciences, Toyama, Japan; ^2^ Research Center for Idling Brain Science, University of Toyama, Toyama, Japan; ^3^ Department of Preventive Intervention for Psychiatric Disorders, National Institute of Mental Health, National Center of Neurology and Psychiatry, Tokyo, Japan; ^4^ Department of Psychiatry, National Center of Neurology and Psychiatry Hospital, Tokyo, Japan; ^5^ Japan Health Research Promotion Bureau, Tokyo, Japan

**Keywords:** cognitive function, depression, erythrocyte membrane, monounsaturated fatty acid, polyunsaturated fatty acid, nervonic acid, oleic acid, schizophrenia

## Abstract

**Introduction:**

Membrane phospholipid abnormalities are considered a pathophysiological background for schizophrenia. The aim of the study was to explore in detail the fatty acid (FA) composition in patients with antipsychotic-free schizophrenia and its association with clinical symptoms and cognitive function.

**Materials and methods:**

Erythrocyte membrane FAs were measured in 29 antipsychotic-free patients with schizophrenia (male/female = 11/18; mean [standard deviation] age=26.7 [7.9] years) and age and sex-matched 32 healthy volunteers. Clinical symptoms and cognitive function were assessed using the Positive and Negative Syndrome Scale (PANSS), Brief Assessment of Cognition in Schizophrenia (BACS), and the Schizophrenia Cognition Rating Scale (SCoRS).

**Results:**

Eicosapentaenoic acid levels were lower in the schizophrenia group than in the healthy control group. In contrast, arachidonic acid and nervonic acid levels were higher in the schizophrenia group than in the control group. Nervonic acid levels were significantly associated with depression scores as measured by the PANSS. No FA levels were correlated with BACS score; however, oleic acid levels were significantly related to cognitive dysfunction, as measured by the SCoRS.

**Conclusion:**

These findings suggest that depressive symptoms along with cognitive dysfunction in daily living in schizophrenia may be linked to the FA composition abnormalities. Further studies will be needed to examine potential longitudinal FA changes during the course of schizophrenia as well as disease specificity.

## Introduction

It has been suggested that the altered composition of phospholipids (composed of the sphingosine skeleton, fatty acid (FA), phosphoric acid, and alcohol), a major component of neural membranes, may be related to the pathophysiology of schizophrenia (1–4). As the phospholipid components likely influence the fluidity and elasticity of cell membranes (5, 6), their changes may lead to an increased membrane rigidity and consequently alter the conformation and function of proteins, receptors, and ion channels (7). Among the constituents of phospholipids, saturated FA e.g. palmitic acid (PA) and stearic acid (SA), as well as monounsaturated FAs (MUFAs), e.g. oleic acid (OA) and nervonic acid (NA), can be synthesized *de novo*. Conversely, some polyunsaturated fatty acids (PUFAs), e.g. n-3 (omega-3) and n-6 (omega-6) PUFAs are called essential unsaturated fatty acids because they must be acquired from the diet. n-3 PUFAs contain eicosapentaenoic acid (EPA), docosapentaenoic acid (DPA), and docosahexaenoic acid (DHA). n-6 PUFAs contain linoleic acid (LA), dihomogammalinolenic acid (DGLA), and arachidonic acid (AA). PUFAs play important roles in the regulation of neuronal migration, pruning, and synaptic plasticity (8). Moreover, n-6 PUFAs are pro-inflammatory bioactive lipids, whereas n-3 PUFAs are anti-inflammatory bioactive lipids (9–11). In this context, n-6/n-3 ratio has been frequently used, since it has been found to be related to several negative health consequences, such as promotion of chronic inflammation (12, 13). Meanwhile, OA can also exert anti-inflammatory effects (14) and NA can activate the antioxidant system (15), these are considered to compensate for deficiencies in essential FAs and their metabolites.

While not consistently replicated, decreased n-3 PUFAs, alteration of n-6 PUFAs and increased n-9 MUFAs in the erythrocyte membrane have been observed in patients with schizophrenia and related conditions (4, 7, 16–20). Several but no all studies also demonstrated an association between PUFAs deficiency and the severity of the negative symptoms of schizophrenia (21–23). Sumiyoshi et al. reported that a decrease in n-3 and n-6 PUFA levels was associated with impaired social cognition in chronically ill patients with schizophrenia (24), while we failed to replicate such relationship in anti-psychotic free first-episode patients (25). Recent studies suggested similar FA abnormalities in at-risk mental state (ARMS) individuals (8, 25) irrespective of outcome (i.e., later psychosis onset), where FA levels were associated with prodromal symptomatology and global functioning. These findings may suggest the role of erythrocyte membrane FA abnormalities as a biomarker associated with vulnerability to psychopathology, but reported FA findings in schizophrenia might also be affected by other factors such as environmental factors after the onset (e.g., dietary habits, physical condition), antipsychotic medication, and chronic oxidative stress. Therefore, further research in patients with less confounding factors is needed to better understand the pathophysiological role of membrane FA composition and its relation to clinical characteristics in schizophrenia.

As human brain is generally inaccessible for direct FA analysis *in vivo*, alternative approaches such as peripheral cell models and neuroimaging techniques have been used as surrogate biomarkers of membrane FAs in the central nervous system (16). Peripheral cell models, especially those of erythrocyte membranes, are frequently used to study brain lipid metabolism due to high correlation between peripheral erythrocyte membrane and brain tissue FA levels (*r* = 0.86) (26). Erythrocyte membrane FA composition is, as compared to plasma FA levels, less affected by food intake prior to blood sampling as a marker of long-term lipid storage (27–29) and also better reflects neural cell membrane FAs in human (30–32) and animals (33, 34).

This study comprehensively measured erythrocyte membrane FAs in antipsychotic-free patients with schizophrenia and healthy controls. The FAs assessed in this study included saturated FAs (PA and SA), n-9 MUFAs (OA and NA), n-3 PUFAs (EPA, DPA, and DHA), and n-6 PUFAs (LA, DGLA, and AA). These FAs were essential for a comprehensive analysis because of their importance as major constituents, accounting for more than 90% of the total FA content in erythrocytes and the brain (5, 18). Based on previous findings, we predicted that patient group would have an altered FA composition (especially decreased n-3 PUFA and increased n-9 MUFA) and that such alterations would contribute to their symptom severity and cognitive functions.

## Methods

### Participants

Twenty-nine Japanese patients with schizophrenia recruited from the University of Toyama Hospital, participated in this study. None of the patients took antipsychotic medications within the two weeks before blood sampling, and 20 of the 29 patients were antipsychotic-naïve. Eleven of the 29 patients had first-episode schizophrenia, defined as an illness duration of fewer than two years with a single psychotic episode. Thirty-two healthy volunteers were recruited from university students, hospital staff, and acquaintances. The patients diagnosed with schizophrenia underwent diagnostic interviews using the Structured Clinical Interview for DSM-IV Axis I Disorders (SCID-I) Patient Edition (35). Eleven of the 29 patients with schizophrenia and all the healthy volunteers overlapped with the subjects in our previous report (25).

Information on clinical history was collected through interviews with the patients, their families, or medical records. Physical examinations and standard laboratory tests confirmed that the participants were physically healthy. The exclusion criteria included being 40 years of age or older, a history of substance abuse or dependence, seizures, head injury, and an estimated premorbid IQ of less than 70 based on the Japanese Adult Reading Test (JART) (36). The JART is the Japanese version of The National Adult Reading Test (Nelson, 1982). It comprises several irregular Japanese words, and the participants’ premorbid IQ is estimated based on their reading performance (36). Additional criteria for healthy controls were; i) no Axis I disorders based on the SCID-I Non-patient Edition (35), and ii) no personal or family (within first-degree relatives) history of psychiatric disorders.

This study was conducted in accordance with the principles of the Declaration of Helsinki and approved by the Committee on Medical Ethics of Toyama University (no. I2013006) on February 5, 2014. Written informed consent was obtained from all participants after a full explanation of the purpose and procedure of the study was provided. Written consent was obtained from the parents or guardians of participants under 20 years of age.

### Clinical assessment

Experienced psychiatrists or psychologists evaluated clinical symptoms, cognitive function, and social function using the Positive and Negative Syndrome Scale (PANSS) (37), Brief Assessment of Cognition in Schizophrenia (BACS) (38, 39), Schizophrenia Cognition Rating Scale (SCoRS) (40, 41), and the modified Global Assessment of Functioning (mGAF) (42). The BACS composite scores were obtained by averaging the z-scores of the six subtests (39). Socio-economic status (SES) was measured by the Hollingshead-Redlich scale (43). Clinical assessments were performed on the same day as or within two weeks of blood collection.

### FA analysis

Blood samples were collected from the study participants between 08:30 and 10:00 after at least two hours of fasting for FA measurements and general blood and biochemical examinations. Erythrocyte membrane FA levels were analyzed by gas chromatography based on an established method (18, 24, 25, 44). Briefly, 1 mL of red blood cells obtained from the subjects was collected in a 15-mL screw cap vial. Erythrocytes and plasma were separated by centrifugation and only erythrocytes were extracted and washed with saline. The vial was filled with 4.0 mL of 0.6 N methanolic HCl containing 4 μL of 0.5% butyl hydroxytoluene (BHT) as an internal standard and was then sealed and incubated at 80°C for two hours. Methylated FAs were extracted twice with hexane and the layers were separated by centrifugation in a swinging rotor at 3000 g for 15 min at room temperature. The hexane layer was carefully removed and the residue was collected in separate vials. The hexane extract was dried entirely by passing it through argon and it was then stored at -40°C until use. The methylated FAs were resuspended in 150 μL hexane, and aliquots (1 μL) were used for FA analysis by a Shimadzu gas chromatograph (Model GC-2010, Japan), using a capillary column with dimensions 30 m×0.32 mm×0.20 μm (Supelco, USA). A flame ionization detector was used with a column oven temperature of 160°C for 10 min, programmed at 10°C rise/min up to 175°C, and held at 220°C for 10 min was used. The injector and detector temperatures were set to 240°C and 275°C, respectively. The column was calibrated by injecting standard FA mixtures in approximately equal proportions. Peaks in the recorded data were identified based on the retention times of standard FAs under identical conditions.

The FA data were categorized into four groups: i) saturated FAs (PA and SA), ii) n-9 series MUFAs (OA and NA), iii) n-3 PUFAs (EPA, DPA, and DHA), and iv) n-6 series PUFAs (LA, DGLA, and AA). The FA levels were expressed as relative values measured as 100% of the 11 FAs, which included the 10 FAs mentioned above, with BHT as an internal standard (5). We calculated the following parameters as indices to assess the inflammatory response: i) n-6/n-3 ratio (AA/[EPA + DHA]) and ii) omega-3 index (EPA + DHA), based on previous literature (8, 45, 46).

### Statistical analysis

Statistical analyses were performed using the Statistical Package for Social Sciences version 25 (SPSS Japan Inc.) and Jamovi Software (https://www.jamovi.org). The analyses covered FA composition and the PANSS, BACS and SCoRS scores and their subscale scores. The mGAF was measured as it is a well-known tool for determining the level of functioning of patients with general mental illness, however was not included in the present analysis due to its strong correlation with SCoRS. As most demographic/clinical data (age, PANSS subscale scores, BACS, SCoRS) had skewed distributions, the nonparametric Mann-Whitney U test was used to compare group differences. Similarly, nonparametric tests were employed to determine group differences in the FA composition, which had non-normal distributions. Spearman’s rho with semi-partial correlation was used to calculate the correlation between FA composition and clinical data, with only FA indices being controlled for by age, because age significantly affected FA composition in previous literature (47, 48) as well as our data (data not shown). For correlation analyses between FA composition and clinical variables, the Benjamini–Hochberg false discovery rate (FDR) procedure was used because there were many items to be compared (49). Statistical significance was set at *p* less than 0.05.

## Results

### Subjects’ profile

Demographic data are shown in **Table 1**. The age and sex ratios of the groups were matched; however, there were significant differences in personal/parental socioeconomic status (controls > patients with schizophrenia). Body mass index (BMI) was higher in the schizophrenia than in the healthy group; however, it was within the normal range (18.5–25) in both groups. The estimated premorbid IQ measured by the JART was within the normal range. Some patients with schizophrenia were taking anxiolytics (n=2) and hypnotics (n=2). These medications did not affect clinical or cognitive indices, or the FAs composition (data not shown). None were taking antidepressant. Regarding cognitive function, patients with schizophrenia performed approximately one standard deviation lower on the composite score calculated from the BACS than those in the healthy group. SCoRS scores were high in the current dataset and exceeded the reported average of first-episode and chronic schizophrenia (3.8 ± 1.8 and 4.4 ± 1.9, respectively) (41). Social functioning measured by the mGAF was approximately 30 points, indicating major impairment in several areas of functioning (42).

**Table 1:** Demographic and clinical data.

	H	Sch	Group difference^a^
	n=32	n=29	
Age [years]	26.9(3.4)	26.7(7.9)	U_32,29 = _439, p=0.72
Gender [male/female]	17/15	11/18	χ^2 = ^1.41, p=0.23
Age at onset [years]	–	23.2(6.9)	–
Duration of illness [years]	–	3.5(5.9)	–
Socioeconomic status	6.6(0.6)	4.4(1.2)	U_32,29 = _49, p<0.001
Parental Socioeconomic status	6.3(0.9)	5.1(0.9)	U_13,24 = _53.5,p<0.001
BMI [kg/m^2^]	20.2(1.6)	22.7(3.4)	U_13,28 = _105, p=0.03
JART	–	100.5(10.2)	–
PANSS	–		–
:positive	–	15.5(5.7)	–
:negative	–	16.3(6.7)	–
:general psychopathology	–	33.3(8.8)	–
:total	–	65.7(17.3)	–
mGAF^b^	–	34.4(10.8)	–
BACS^c^	–	-1.1(1.1)	–
SCoRS^d^	–	6.4(2.3)	–

Values are shown as means (standard deviations).

BACS; Brief Assessment of Cognition in Schizophrenia, BMI; body mass index, H; healthy control, JART; Japanese Adult Reading Test, mGAF; modified Global Assessment Functioning, PANSS; Positive and Negative Syndrome Scale, Sch; schizophrenia, SCoRS; Schizophrenia Cognition Rating Scale.

^a^Demographic differences between groups were examined by Mann-Whitney U t-test or chi-square (χ^2^) test. The subscript two numbers written after U are the sample size in each group.

^b^Data are ranging from 0 to 100. Healthy subjects generally have a score ranging from 90 to 100.

^c^BACS composite score was calculated by averaging all z-scores of the six primary measures from the BACS.

^d^Data are ranging from 0 to 10, with larger number representing more worse function.

### FA composition

**Figure 1:**
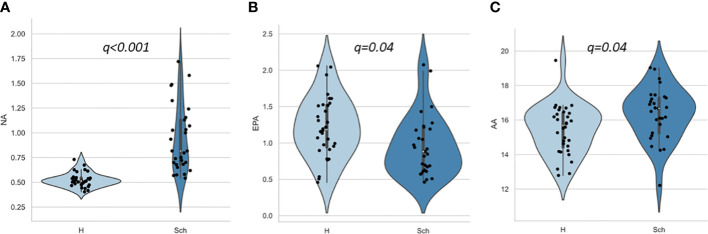
Scatter and violin plots of fatty acid composition. **(A)**, **(B)**, and **(C)** represent NA, EPA and AA compositions for H and Sch, respectively. The y-axis shows the percentage of each fatty acid when all (ten) fatty acids plus the internal standard, are adjusted to 100%. Values indicate false discovery rate adjusted p value (*q*). AA, arachidonic acid (20:4 n-6); EPA, eicosapentaenoic acid (20:5 n-3); H, healthy control; NA, nervonic acid (24:1 n-9); Sch, schizophrenia.

**Table 2** and **Figure 1** present the results of FA composition analysis. The NA levels were significantly higher in the schizophrenia group than in the healthy group. EPA levels were lower and AA levels were higher in the schizophrenia group than in healthy controls. There was a significant difference in DPA levels and the n-6/n-3 ratio between controls and patients with schizophrenia; however, this did not persist after post-hoc analysis for multiple comparisons. These findings remained consistent even when we analyzed only antipsychotic-naïve or only first-episode patients (data not shown).

**Table 2:** Fatty acid composition.

		H	Sch	Statistic	*p*	*q*	effect size
		n=32	n=29				
saturated	PA	21.17 (0.98)	21.17 (1.01)	U_32,29_=439	0.72	0.79	0.05
	SA	19.80 (0.65)	19.56 (0.99)	U_32,29_=400	0.36	0.48	0.14
monounsaturated	OA	14.69 (1.09)	14.72 (1.03)	U_32,29_=455	0.90	0.90	0.02
	NA	0.53 (0.07)	0.94 (0.33)	U_32,29_=39	<0.001	<0.001*	0.92
n3 polyunsaturated	EPA	1.22 (1.42)	0.96 (0.42)	U_32,29_=287	0.01	0.04*	0.38
	DPA	3.00 (0.35)	2.85 (0.34)	U_32,29_=328	0.05	0.12	0.29
	DHA	8.16 (1.23)	7.94 (1.35)	U_32,29_=385	0.26	0.45	0.17
n6 polyunsaturated	LA	10.22 (0.93)	10.02 (0.96)	U_32,29_=425	0.58	0.70	0.08
	DGLA	1.44 (0.19)	1.51 (0.30)	U_32,29_=399	0.35	0.53	0.14
	AA	15.44 (1.42)	16.36 (1.52)	U_32,29_=280	0.007	0.04*	0.40
Summary value	n6/n3 ratio^a^	1.72 (0.47)	1.92 (0.47)	U_32,29_=326	0.05	0.14	0.30
	Omega3 Index^b^	9.38 (1.55)	8.90 (1.69)	U_32,29_=348	0.10	0.19	0.25

All values are shown as means (standard deviations).

Differences between groups were examined by Mann-Whitney U t-test. The subscript two numbers written after U are the sample size in each group.

*False discovery rate adjusted *p* value (*q*) < 0.05 (bold letter).

AA, arachidonic acid (20:4 n-6); DGLA, dihomogammalinolenic acid (20:3 n-6); DHA, docosahexaenoic acid (22:6 n-3); DPA, docosapentaenoic acid (22:5 n-3); EPA, eicosapentaenoic acid (20:5 n-3); H, healthy control; LA, linoleic acid (18:2 n-6); NA, nervonic acid (24:1 n-9); OA, oleic acid (18:1 n-9); PA, palmitic acid (16:0); SA, stearic acid (18:0); Sch, schizophrenia.

^a^n-6/n-3 ratio=AA/(EPA+DHA).

^b^omega-3 index=EPA+DHA.

### Relationships between FAs and clinical symptoms

**Figure 2:**
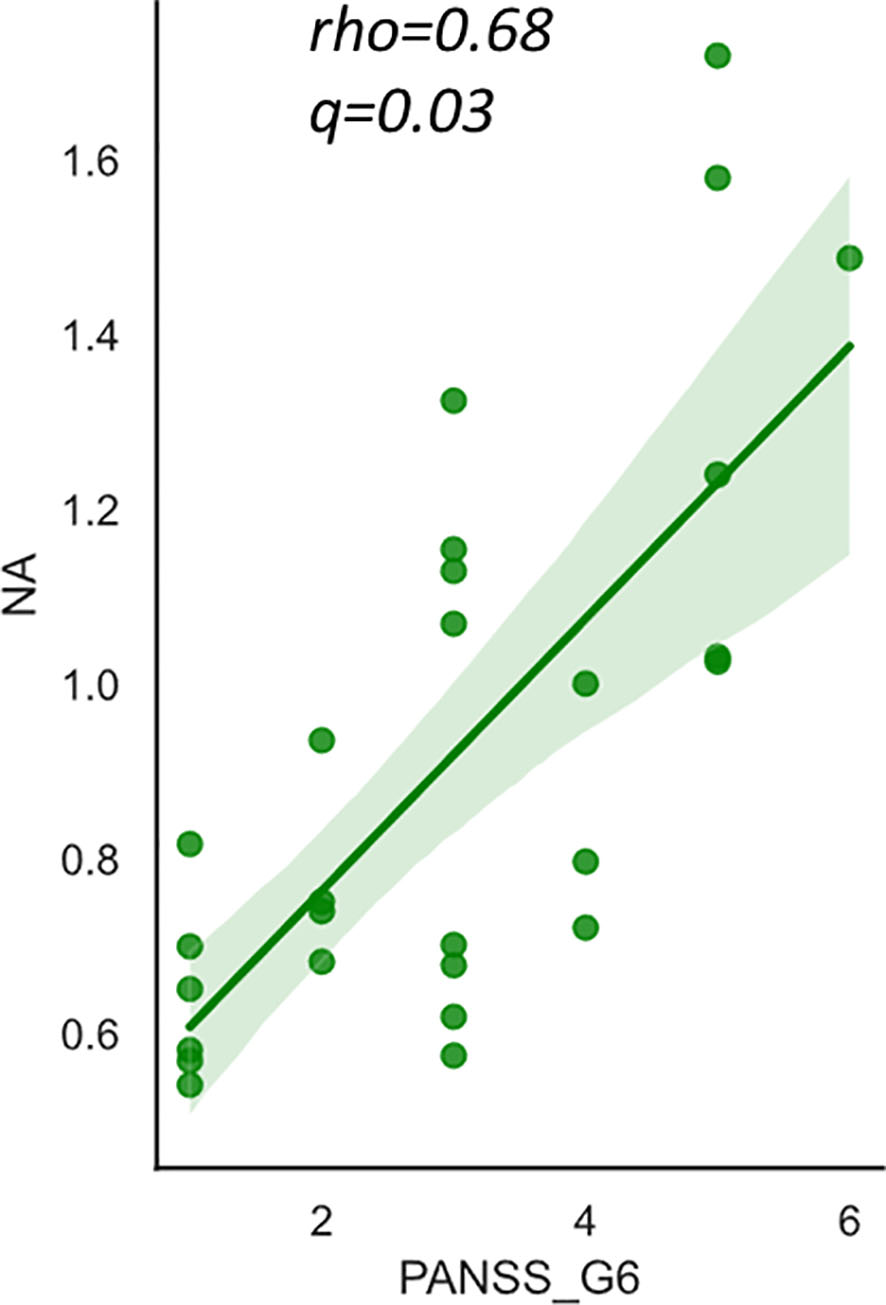
The relationship between nervonic acid (NA) and PANSS_G6 (depression) score in schizophrenia. The y-axis shows the percentage of each fatty acid when all (ten) fatty acids plus the internal standard, are adjusted to 100%. Values represent Spearman’s rho and false discovery rate adjusted p value (q). The shaded area represents 95% confidence interval. NA, nervonic acid (24:1 n-9), PANSS, Positive and Negative Syndrome Scale.

No correlations were found between the FAs and PANSS total or positive/negative scores. Only NA was weakly correlated with PANSS general psychopathology scores (*rho*=0.47, *p*=0.017), however this correlation did not survive after multiple comparisons (**Table 3A**). Regarding the subscales, a significant positive correlation was found between NA level and “depression” score (G6) as presented in **Table 3B** and **Figure 2**. NA level was also correlated with “anxiety” (G2) and “poor attention” (G11) scores, but they did not survive post-hoc analysis for multiple comparisons. There were no significant correlations between the other FAs or the summary values (the n6/n3 ratio and n-3 index) and PANSS subscale scores (data not shown).

Table 3Relationships between Fatty acid concentration and PANSS scores in schizophrenia.

**A. Fatty acid compositions and PANSS total, positive, negative, and general psychopathology:** 

	PANSS total			PANSS Positive			PANSS Negative			PANSS General Psychopathology		
	rho	*p*	*q*	rho	*p*	*q*	rho	*p*	*q*	rho	*p*	*q*
**PA**	0.37	0.07	0.42	0.07	0.73	1.75	0.40	0.05	0.29	0.32	0.12	0.47
**SA**	-0.42	0.04	0.45	-0.02	0.93	1.01	-0.40	0.05	0.54	-0.37	0.07	0.41
**OA**	0.23	0.28	0.67	0.13	0.53	1.59	0.34	0.10	0.39	0.07	0.75	1.12
**NA**	0.35	0.09	0.35	-0.03	0.89	1.18	0.23	0.28	0.67	0.49	0.01	0.16
**EPA**	-0.17	0.41	0.82	-0.15	0.46	1.85	-0.15	0.47	0.95	-0.09	0.65	1.57
**DPA**	-0.27	0.19	0.58	-0.24	0.23	2.75	-0.23	0.27	0.81	-0.17	0.42	1.25
**DHA**	0.02	0.91	1.21	-0.01	0.96	0.96	-0.04	0.85	1.13	0.07	0.74	1.27
**LA**	-0.03	0.90	1.35	0.16	0.43	2.59	-0.14	0.51	0.88	-0.05	0.80	1.07
**DGLA**	0.06	0.80	1.36	0.06	0.76	1.51	-0.03	0.89	1.07	0.03	0.90	0.90
**AA**	0.01	0.96	1.15	-0.05	0.82	1.23	-0.04	0.84	1.27	0.08	0.69	1.38
**n6/n3ratio^a^**	0.00	0.99	0.99	-0.02	0.91	1.09	-0.01	0.95	0.95	0.03	0.88	0.96
**Omega3 Index^b^**	0.00	0.98	1.07	-0.05	0.80	1.37	-0.03	0.90	0.98	0.04	0.87	1.04

**B. NA and PANSS subscales:** 

		rho	*p*	*q*
**PANSS Positive**	**1^c^**	0.03	0.89	0.91
	**2**	0.09	0.67	0.89
	**3**	0.04	0.86	0.89
	**4**	-0.08	0.71	0.38
	**5**	-0.36	0.08	0.89
	**6**	0.06	0.77	0.33
	**7**	0.32	0.11	0.89
**PANSS Negative**	**1**	0.32	0.11	0.37
	**2**	0.17	0.42	0.73
	**3**	0.21	0.32	0.63
	**4**	0.39	0.05	0.41
	**5**	-0.14	0.50	0.78
	**6**	0.23	0.27	0.59
	**7**	-0.34	0.09	0.40
**PANSS General Psychopathology**	**1**	0.39	0.06	0.33
	**2**	0.45	0.02	0.33
	**3**	-0.05	0.81	0.86
	**4**	-0.34	0.10	0.36
	**5**	0.17	0.42	0.78
	**6**	0.68	<0.001	0.03*
	**7**	0.29	0.16	0.43
	**8**	-0.07	0.76	0.91
	**9**	0.11	0.62	0.88
	**10**	0.05	0.80	0.89
	**11**	0.45	0.03	0.25
	**12**	0.28	0.17	0.43
	**13**	0.17	0.42	0.70
	**14**	-0.13	0.53	0.80
	**15**	0.25	0.23	0.53
	**16**	0.09	0.68	0.89

Values are Spearman’s rank correlation coefficient, calculated using semi-partial correlation analysis that only fatty acid was controlled by age as a covariate.

*False discovery rate adjusted *p* value (*q*) < 0.05 (bold letter).

AA, arachidonic acid (20:4 n-6); DGLA, dihomogammalinolenic acid (20:3 n-6); DHA, docosahexaenoic acid (22:6 n-3); DPA, docosapentaenoic acid (22:5 n-3); EPA, eicosapentaenoic acid (20:5 n-3); H, healthy control; LA, linoleic acid (18:2 n-6); NA, nervonic acid (24:1 n-9); OA, oleic acid (18:1 n-9); PA, palmitic acid (16:0); PANSS; Positive and Negative Syndrome Scale, SA, stearic acid (18:0).

^a^n-6/n-3 ratio=AA/(EPA+DHA).

^b^omega-3 index=EPA+DHA.

^c^The numbers represent the following items, respectively:

Positive;1=Delusions, 2=Conceptual disorganization, 3=Hallucinations, 4=Excitement, 5=Grandiosity, 6=Suspiciousness/persecution, 7=Hostility.

Negative; 1=Blunted affect, 2= Emotional withdrawal, 3= Poor rapport, 4= Passive/apathetic social withdrawal, 5= Difficulty in abstract thinking, 6= Lack of spontaneity and flow of conversation, 7=Stereotyped thinking.

General Psychopathology; 1=Somatic concern, 2=Anxiety, 3=Guilt feelings, 4=Tension, 5=Mannerisms and posturing, **6=Depression,** 7=Motor retardation, 8=Uncooperativeness, 9=Unusual thought content, 10=Disorientation, 11=Poor attention, 12=Lack of judgment and insight, 13=Disturbance of volition, 14=Poor impulse control, 15=Preoccupation, 16=Active social avoidance.

### Relationships between FAs and cognitive scales

The relationships between FAs and cognitive scales are presented in **Table 4**. A significant positive correlation was found between OA level and SCoRS score. The BACS composite score correlated with SA level; however, this correlation did not persist after post-hoc analysis for multiple comparisons. There were no significant correlations between the other FAs or summary values (the n6/n3 ratio and n-3 index) and cognitive scales.

**Table 4:** Relationships between fatty acid composition and cognitive and social functions in schizophrenia.

		BACS			SCoRS		
		rho	*p*	*q*	rho	*p*	*q*
saturated	PA	-0.10	0.62	0.99	0.19	0.38	0.83
	SA	0.44	0.02^†^	0.24	-0.37	0.09	0.52
n-9 monounsaturated	OA	-0.32	0.10	0.47	0.60	0.002	0.048*
	NA	-0.37	0.06	0.46	-0.07	0.76	1.02
n-3 polyunsaturated	EPA	-0.17	0.39	0.67	0.04	0.87	0.99
	DPA	-0.19	0.33	0.80	-0.19	0.38	0.76
	DHA	-0.22	0.28	0.74	-0.02	0.94	1.03
n-6 polyunsaturated	LA	0.25	0.20	0.81	-0.19	0.39	0.71
	DGLA	-0.001	0.99	0.99	-0.07	0.75	1.12
	AA	0.06	0.78	0.98	-0.01	0.98	1.02
Summary value	n-6/n-3 ratio^a^	0.22	0.26	0.79	-0.07	0.75	1.06
	omega-3 index^b^	-0.24	0.23	0.78	0.05	0.81	0.97

Values are Spearman’s rho, calculated using semi-partial correlation analysis that only fatty acid indices were controlled by age as a covariate.

*False discovery rate adjusted *p* value (*q*) < 0.05 (bold letter).

AA, arachidonic acid (20:4 n-6); BACS, Brief Assessment of Cognition in Schizophrenia; DGLA, dihomogammalinolenic acid (20:3 n-6); DHA, docosahexaenoic acid (22:6 n-3); DPA, docosapentaenoic acid (22:5 n-3); EPA, eicosapentaenoic acid (20:5 n-3); FES, first episode schizophrenia; H, healthy control; LA, linoleic acid (18:2 n-6); NA, nervonic acid (24:1 n-9); OA, oleic acid (18:1 n-9); PA, palmitic acid (16:0); SA, stearic acid (18:0); SCoRS, Schizophrenia Cognition Rating Scale.

^a^n-6/n-3 ratio=AA/(EPA+DHA).

^b^omega-3 index=EPA+DHA.

**Figure 3:**
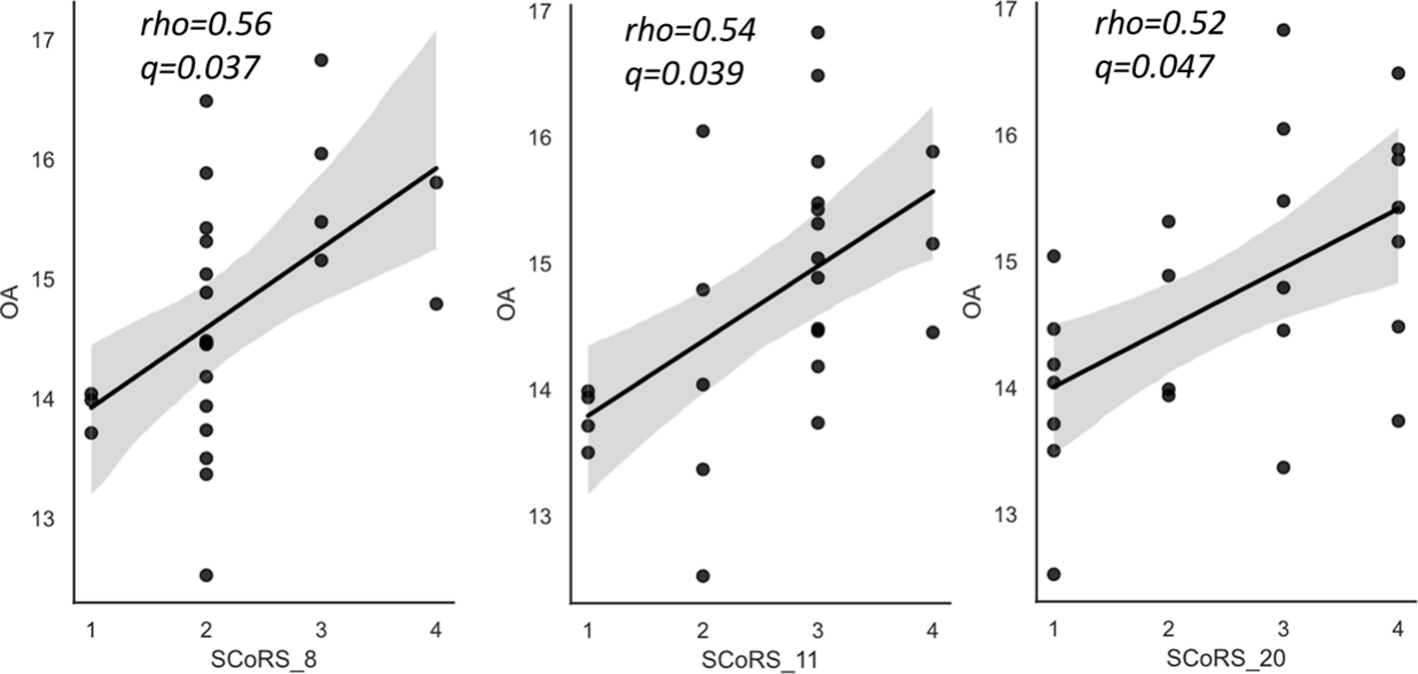
The relationships between oleic acid (OA) and SCoRS subscales in schizophrenia. SCoRS_8; ‘Remembering what you were going to say?’; SCoRS_11, ‘Concentrating well enough to read a newspaper or a book?’; SCoRS_20, ‘Following conversations in a group?’. The y-axis shows the percentage of each fatty acid when all (ten) fatty acids plus the internal standard, are adjusted to 100%. Values represent Spearman’s rho and false discovery rate adjusted p value (q). The shaded areas represent 95% confidence interval. OA, oleic acid (18:1 n-9); SCoRS, Schizophrenia Cognition Rating Scale.

The correlation results between the OA and SCoRS subscales are presented in **Table 5** and **Figure 3**. There were significant positive correlations between OA level and items 8 (remembering what you were going to say)?, 11 (concentrating sufficiently to read newspapers or books)?, and 20 (following conversations in a group)?.

**Table 5:** Relationships between OA concentration and SCoRS subscores in schizophrenia.

SCoRS subscale	rho	*p*	*q*
1. Remembering names of people you know or meet?	0.20	0.36	0.40
2. Remembering how to get places?	0.33	0.13	0.18
3. Following a TV show?	0.31	0.15	0.18
4. Remembering where you put things?	0.41	0.05	0.10
5. Remembering your chores and responsibilities?	0.44	0.04	0.09
6. Learning how to use new gadgets and equipment?	0.07	0.76	0.76
7. Remembering information and/or instructions recently given to you?	0.46	0.03	0.08
**8. Remembering what you were going to say?**	0.56	0.006	0.037*
9. Keeping track of your money?	0.34	0.11	0.18
10. Keeping your words from being jumbled together?	0.35	0.10	0.17
**11. Concentrating well enough to read a newspaper or a book?**	0.54	0.008	0.039*
12. With familiar tasks?	0.25	0.25	0.29
13. Staying focused?	0.56	0.01	0.05
14. Learning new things?	0.49	0.02	0.06
15. Speaking as fast as you would like?	0.33	0.13	0.17
16. Doing things quickly?	0.35	0.10	0.18
17. Handling changes in your daily routine?	0.41	0.05	0.11
18. Understanding what people mean when they are talking to you?	0.56	0.005	0.11
19. Understanding how other people feel about things?	0.08	0.73	0.77
**20. Following conversations in a group?**	0.52	0.01	0.047*

Values are Spearman’s rank correlation coefficient, calculated using semi-partial correlation analysis that only OA was controlled by age as a covariate.

*False discovery rate adjusted *p* value (*q*) < 0.05 (bold letter).

OA, oleic acid (18:1 n-9); SCoRS, Schizophrenia Cognition Rating Scale.

## Discussion

The present study investigated the erythrocyte membrane FA composition and its relationship with symptom severity, social, and cognitive functioning in antipsychotic-free patients with schizophrenia. Our findings showed that patients with schizophrenia had decreased levels of EPA (an n-3 PUFA), increased levels of AA (an n-6 PUFA), and a markedly increased level of NA (an n-9 MUFA), compared to controls. We also found that NA levels were predominantly associated with depression and related symptoms in patients with schizophrenia. Moreover, OA, an n-9 PUFA, was significantly and positively related to cognitive dysfunction related to daily living, as measured by the SCoRS in schizophrenia. These results suggest an association with specific aspects of symptomatology, suggesting FA composition may contribute.

The decreased EPA level in the current schizophrenia group is consistent with previous research (4, 7, 18), indicating that this change may be a characteristic trait of schizophrenia. FA levels including EPA composition are known to be affected by various confounding factors, such as antipsychotics (5, 7, 23, 50–53), aging (47, 48), dietary intake (54), and smoking (54). For this reason, this study examined only antipsychotic-free schizophrenia patients with early illness stages matched for age with control subjects, who likely have fewer confounding factors compared to previous studies. We also observed increased levels of AA, an n-6 PUFA. Previous studies consistently found an Increased n-6/n-3 ratio in schizophrenia (4, 5, 20). AA-derived eicosanoids have more prominent inflammatory activity than n-3 PUFAs, and an imbalance between n-6 and n-3 PUFAs, as indicated by the increased n-6/n-3 ratio, may cause neuroinflammatory pathology in neuropsychiatric disorders (55, 56). In other words, AA is a precursor of inflammatory bioactive lipids such as prostaglandins and thromboxane, which act in a pro-inflammatory direction (9, 10). Conversely, EPA is a known precursor of anti-inflammatory bioactive lipids, particularly resolvins E1/2, which are potent anti-inflammatory mediators and act in the direction of converging inflammation (11). Our findings suggest that patients with schizophrenia may have an excess of induced inflammation, resulting in damage to neurons or other organs. Furthermore, animal and experimental studies have suggested that PUFA abnormalities affect membrane properties in the central nervous system (e.g., fluidity, elasticity, and thickness) (6) and dopaminergic transmission (57), which might underlie vulnerability to psychosis (58). A combination of these factors may shape the pathophysiology of schizophrenia.

The role of PUFAs in the pathophysiology of schizophrenia led to the hypothesis that n-3 PUFAs (EPA and DHA) could be administered to patients with ARMS to prevent the onset of overt psychosis. Accordingly, the worldwide multicenter RCT, NEURAPRO, was conducted (59). However, n-3 PUFA did not provide additional benefits for psychosis prevention. Considering this finding, the role of n-3 PUFAs in schizophrenia remain unclear, suggesting that the changes observed in the present study also might be a secondary phenomenon rather than pathophysiology of schizophrenia. Conversely, a recently published network meta-analysis concluding that n-3 PUFAs helped in preventing transitions to psychosis as compared to in controls (60). Currently, Other large-scale studies have been conducted (https://clinicaltrials.gov/ct2/show/NCT01429454; https://clinicaltrials.gov/ct2/show/record/NCT02597439), and their conclusions are expected to further our understanding of the role of FAs in schizophrenia.

Another important finding in this study was a markedly high level of NA, an n-9 MUFA, in the schizophrenia group. This finding is consistent with previous studies that indicated increased NA levels in patients with schizophrenia and ARMS (16, 20, 25). We also found a positive correlation between NA and depressive symptoms as measured by the PANSS subscale. Anxiety and poor attention, which are common symptoms with patients with depression (61, 62) also showed trend-level correlation with an increased NA level. NA is an essential molecule for the growth and maintenance of integrity in the white matter and peripheral nervous tissue enriched by sphingomyelin (63) and is suggested to be related to psychiatric disorders (64). Our results may be partly in line with the neuroimaging evidence that abnormalities in brain connectivity contribute to the trait characteristics of schizophrenia (65), such as negative symptomatology (66) and cognitive deficits (67). Because the impaired integrity of white matter is also reported in depression (68–70), it may be possible that NA abnormalities commonly contribute to the pathophysiology of both schizophrenia and depression.

In this study, we found for the first time that OA and n-9 MUFA levels were significantly related to cognitive dysfunction related to daily living, as measured by the SCoRS, in patients with schizophrenia. As far as we know, no previous study has shown significant association between OA levels and schizophrenia; however, previous OA studies have shown associations with depression (71, 72), Parkinson’s disease (73) and Alzheimer’s disease (74). Notably, a recent large-scale study on depression (n=4459) (75) suggested that serum OA levels were positively associated with depression and that for every 1 mmol/L increase in OA levels, the prevalence of depression increased by 40%. OA is an important FA as the most abundant FA in plasma, accounting for approximately 80% of the plasma phospholipid MUFAs (76). Since stearoyl-CoA desaturase, the rate-limiting enzyme of OA, has been found to cause neurotoxicity by producing MUFAs and impairing microglia and macrophages (77), the possibility exists that this mechanism affects cognitive function in patients with schizophrenia.

The present study had several potential limitations. First, the sample size of the study was rather small by including only antipsychotic-free patients, which may have limited the generalizability and statistical power of our results. Second, we did not control for participants’ dietary habits and cigarette smoking that could affect FA results. All participants had normal blood test results and standard BMIs; however, the BMIs were higher in the patients than in the controls, whereas the SES was higher in the controls than in the patients. The SES score did not always reflect the exact nutritional status of the young patients included in this study; thus, we should have conducted a nutritional questionnaire survey in addition to the assessments of SES, BMI, and blood chemistry. Differences in the nutritional status potentially led to differences in the lifestyle habits and physical conditions between the groups. The possibility that this further affected the results of the symptom/cognition measures in each group cannot be ruled out. Third, as our study was cross-sectional, future longitudinal studies are needed to confirm the role of FA changes as a trait marker and to investigate the influence of illness stage. Finally, as FA abnormalities and their contribution to clinical symptoms were reported also in other neuropsychiatric disorders, such as depression ^[63]^, disease specificity of our findings should be tested in future studies.

In conclusion, this study expanded our previous FA findings in schizophrenia and confirmed the increase in EPA levels and decrease in AA levels in the affected patients. It further showed that erythrocyte membrane level of NA, an n-9 MUFA elevated in patients with possibility of impaired white matter integrity (65, 68–70), was significantly correlated with the severity of depressive symptoms in a cohort of antipsychotic free patients with relatively early stages. We also demonstrated for the first time that membrane OA, an n-9 MUFA abundantly exists in the human body, is associated with cognitive dysfunction in schizophrenia. Further studies on FA abnormalities in various illness stages of schizophrenia, potential influencing factors, and disease specificity will be needed to clarify the role of FA abnormalities in the pathophysiology of schizophrenia.

## Data availability statement

The raw data supporting the conclusions of this article will be made available by the authors, without undue reservation.

## Ethics statement

The studies involving humans were approved by The Committee on Medical Ethics of the University of Toyama. The studies were conducted in accordance with the local legislation and institutional requirements. Written informed consent for participation in this study was provided by the participants’ legal guardians/next of kin.

## Author contributions

YH: Conceptualization, Data curation, Formal analysis, Funding acquisition, Investigation, Methodology, Project administration, Resources, Software, Supervision, Validation, Visualization, Writing – original draft, Writing – review & editing. TT: Data curation, Funding acquisition, Investigation, Project administration, Resources, Supervision, Writing – review & editing. HI: Data curation, Investigation, Writing – review & editing. DS: Data curation, Funding acquisition, Investigation, Project administration, Resources, Supervision, Writing – review & editing. TS: Conceptualization, Data curation, Formal analysis, Investigation, Methodology, Project administration, Resources, Supervision, Writing – review & editing. MS: Conceptualization, Formal analysis, Funding acquisition, Investigation, Methodology, Project administration, Resources, Supervision, Writing – review & editing, Data curation, Validation.
